# Treatment of Disease by Oxygen

**Published:** 1866-09

**Authors:** 


*Treatment of Disease by Oxygen.*

In the Lancet, March 10, 1866, Dr. R. H. Goolden furnishes the results of some experiments he has been carrying on at St. Thomas Hospital, London, on the application of oxygen as a remedial agent in the treatment of disease.

The idea of inhaling oxygen was mooted by Priestly soon after its discovery in 1774, and by Sir Humphrey Davy and by Dr. Beddoes in 1804, but not adopted to any extent. Until Dr. Goolden could obtain a proper instrument he used the oxygen water and the binoxide of hydrogen. The latter he exhibited in one drachm doses, diluted in two ounces of water. He found it to have a marked influence on the biliary secretion, increasing the quantity and improving the quality, and often producing large biliary dejections. At the same time he causes his patient to inhale a mixture of oxygen and air, in the proportion of 1 to 4, from a large vulcanite bag, with a tube, stop-cock and mouthpiece. Its use is continued for half an hour daily, slowly inspiring at intervals, and filling the lungs as much as possible.

In chronic gout Dr. Goolden has seen this treatment followed by clear urine, great relief, and in some cases cures have resulted. He has latterly found, in carefully selected cases, the Turkish bath a great expeditor of the absorption of enlarged joints, and a valuable adjunct to the oxygen treatment, and that in cases where

Turkish bath alone had failed. Lithic acid does not appear in the cutaneous excretion of the Turkish bath, even where it is known to abound in the blood.

Dr. Demarquay, in a recent work, "*Essai de Pneumatologie Medicale*," treats of the practical value and effects of the inhalation of oxygen. At first the inhalation of oxygen produces a sensation of heat in the mouth, which soon ceases; the skin becomes warm, and sometimes slightly moist; the pulse is quickened, and becomes harder; the appetite returns, with a desire for muscular exercise.

In incipient phthisis, before fever comes on, and the patient is rapidly emaciating, with persistent indigestion, oxygen has a salutary influence. In anæmia, particularly in the chlorosis of young girls, with its attending obstinate anorexia, in the anæmia of persons convalescing from acute diseases and following hemorrhages, and especially in that variety met with in the puerperal state, oxygen is a valuable remedy. Where persons are debilitated, by long and profuse suppuration, it is useful. In all these affections inhalations of oxygen seem to sustain the forces of the patient, and help towards recovery.

In surgery, oxygen baths may be used to improve the state of slowly healing and ill conditioned wounds and ulcers, and to hasten cicatrization. The observations of Lagier, Maurice Raynaud and Demarquay, leave no doubt of its value in senile gangrene of the foot, so long as the circulation is maintained in the plantar artery.

Dr. F. Bricheteau (Bulletin Gen. de Therapeutique Med. et Chir., Feb. 28, 1866,) has published a paper on the "Therapeutic Employment of Oxygen Gas," in which very minute instructions are given for its administration, with a description of the apparatus of Limousin, of Paris, for the inhalation of oxygen. The gas should be prepared invariably by the decomposition of the chlorate of potash. (*Preparation du Gaz Oxygene pour Inhalations*, par M. Limousin, Pharmacien, *Bullet. de Therap.*, t. lxviii., p. 167.) It is then introduced into a caoutchouc or silk bag, with a flexible tube, which, furnished with two stop-cocks, terminates in a straight glass tube introduced into a water goblet, half filled with water; a second pipe passes through the cork in the mouth of the goblet and to it is attached another flexible tube, furnished with an ivory mouthpiece. The oxygen, either pure or diluted, passes through the water, and is washed from all impurities it may have. The dose varies with the age and condition of the patient, but ordinarily from thirty-five to fifty pints are given in the course of the day, half in the morning and half in the evening. Even when pure oxygen is put into the receiver, a certain amount of atmospheric air is necessarily respired at the same time through the nostrils. Dr. Bricheteau adds his testimony to the quick and decided improvement of the appetite under the use of inhalation of oxygen. Berenger-Feraud (*Bullet. de Therap.* t. lxvii.) has shown satisfactorily the great diminution in the amount of sugar in the urine of diabetic patients during its use.

The testimony so far given in favor of the therapeutic employment of oxygen gas by inhalations and local baths, warrants its admission as an article of the materia medica, and its rational employment in practical medicine.—*The New York Medical Journal.*

